# Determinants of acute undernutrition among pregnant women attending primary healthcare unit in Chinaksen District, Eastern Ethiopia: a case-control study

**DOI:** 10.7717/peerj.15416

**Published:** 2023-06-05

**Authors:** Hassen Abdi Adem, Ahmedin Aliyi Usso, Habtemu Jarso Hebo, Abdulhalik Workicho, Fila Ahmed

**Affiliations:** 1 School of Public Health, College of Health and Medical Sciences, Haramaya University, Harar, Harari, Ethiopia; 2 School of Nursing and Midwifery, College of Medicine and Health Sciences, Jigjiga University, Jigjiga, Somali, Ethiopia; 3 Department of Epidemiology, Faculty of Public Health, Jimma University, Jimma, Oromia, Ethiopia

**Keywords:** Determinants, Undernutrition, Pregnant women, Case-control study, Ethiopia

## Abstract

**Background:**

Women’s undernutrition during pregnancy increases the risks and burdens of maternal and neonatal morbidity, death, and disability through its vicious cycles of irreversible intergenerational negative effects. Despite the high burden of maternal undernutrition during pregnancy in semi-pastoral communities of eastern Ethiopia, there is a paucity of information on its major risk factors. This study revealed determinants of acute undernutrition among pregnant women attending primary healthcare units in Chinaksen district in rural eastern Ethiopia.

**Method:**

A facility-based case-control study was conducted among 113 cases and 113 controls in Chinaksen district from February 01 to March 30, 2017. Data were entered using EpiData version 3.1 and analyzed using SPSS version 24. Multivariable logistic regression analyses conducted to identify significant determinants of acute undernutrition. Adjusted odds ratio (AOR) with a 95% confidence interval was used to report the strength of association and statistical significance declared at *p* value < 0.05.

**Results:**

Sixty (53.1%) of cases and 56 (49.6%) of controls were in the age group of 25-34 years and their mean ± SD age of cases and controls were 26.6 ± 5.7 and 28 ± 5.5 years, respectively. In this study, larger family size (AOR = 6.98, 95 CI [2.82–17.27]), lack of prenatal dietary advice (AOR = 3.68, 95% CI [1.67–8.00]), did not participate in a cooking demonstration (AOR = 5.41, 95% CI [2.39–12.24]), used substances (AOR = 3.65, 95% CI [1.30–10.23]), absence of basic latrine (AOR = 2.91, 95% CI [1.28–6.58]), low minimum dietary diversity of women (AOR = 2.48, 95% CI [1.20–5.12]), and household food insecurity (AOR = 3.06, 95% CI [1.44–6.51]) were significantly increased the odds of acute undernutrition among pregnant women.

**Conclusions:**

The study revealed that living in crowded families, lack prenatal dietary advice, did not participate in cooking demonstrations, substances use; lack of toilet, low minimum dietary diversity, and household food insecurity were significant risk factors for acute undernutrition among pregnant women. Strengthening multi-sectoral approaches through improving dietary diversity/quality and food access/quantity would be essential to prevent, and reduce the risks, burdens, and impacts of maternal undernutrition during pregnancy.

## Introduction

Maternal undernutrition is a worldwide major public health problem (Ahmed, Hossain & Sanin, 2012). It affects estimated 462 million women in the world (Zahangir et al., 2017) and 240 million women in low and middle-income countries (LMIC), each year (NCD-RisC, 2016). The maternal undernutrition rate ranges from 10–19% globally (Ahmed, Hossain & Sanin, 2012; WHO, 2017; Zahangir et al., 2017), and more than 20% in LMIC (Black et al., 2013; Tang et al., 2013; Urooj, Rao & Sesikeran, 2018), and 27–51% in Sub-Saharan Africa (SSA) (Branca et al., 2015; Tang et al., 2013; Urooj, Rao & Sesikeran, 2018; WHO, 2017). The burden of maternal undernutrition is also high in Ethiopia, ranging from 9.2%–47.9% (Abraham, Miruts & Shumye, 2015; Central Statistical Agency Ethiopia and ICF, 2016; Desalegn et al., 2015; Federal Ministry of Health, 2012; Mathewos, Amare & Eskindir, 2015; Shiferaw & Husein, 2019).

Maternal nutritional status during pregnancy is the major determent of maternal, neonatal, and child health outcomes (Urooj, Rao & Sesikeran, 2018). Poor maternal nutritional status during pre-pregnancy and/or pregnancy increases the risk of adverse pregnancy outcomes and birth complications such as low birth weight, preterm birth, intrauterine growth retardation, obstructed labor, anemia, cesarean delivery, and antepartum and postpartum hemorrhages (Adebowale et al., 2011; Cates et al., 2017; WHO, 2016). For instance, globally estimated 20.5 million newborns were delivered with low birth weight each year and of this number, 48% (9.84 million) were from Southeast Asia, 24% (4.92 million) were from SSA (Blencowe, Krasevec & De Onis, 2019), and 17% (3.49 million) from Ethiopia (Endalamaw et al., 2018). Moreover, maternal undernutrition during pregnancy directly or indirectly increases the risks of maternal, neonatal and child morbidity, mortality and disability (WHO, 2016). For example, it increases maternal death by 20% in the world (USAID, 2014) and by 23% in developing countries (Black et al., 2013).

On the other hand, women’s undernutrition during pregnancy has negative economic effects. For example, it reduces the gross domestic product of a given country by 1.4 to 2.1 trillion US dollars per year globally (WHO, 2016) and 3–16% of gross domestic product per year in Africa, and 16.5% of its gross domestic product per year in Ethiopia (UN Economic Commission for Africa, 2014; Ministry of Health, Ethiopia, 2016).

Despite the several intervention measures undertaken to prevent maternal undernutrition during pregnancy and reduce the burdens of the problem in SSA including Ethiopia (Central Statistical Agency Ethiopia and ICF International, 2012), the prevalence of maternal undernutrition is slowly declined by eight points in the last two decades, from 30.5% in 2000 to 22% in 2016 (Central Statistical Agency Ethiopia and ICF, 2016). A previous study indicated that one in every five pregnant women was undernourished in rural eastern Ethiopia (Kedir, Berhane & Worku, 2016).

Few previous studies indicated that dietary factors (such as poor food intake and poor diet quality), infections, inter-birth interval, demographic factors (such as age, marital status, occupational status, and educational status) and economic factors (wealth index) were the factors associated with acute undernutrition of pregnant women (Abraham, Miruts & Shumye, 2015; Ayana, Hailemariam & Melke, 2015; Davies et al., 2012).

Previous risk factors studies conducted were focused on children (Abebaw & Zewdie, 2013; Ayana, Hailemariam & Melke, 2015) and non-pregnant women (Abraham, Miruts & Shumye, 2015) and addressed the burden of maternal undernutrition during pregnancy (Kedir, Berhane & Worku, 2016), yet there is a paucity of information on its preventable major determinants. In addition, certain previous studies used Body Mass Index (BMI) <18.5 kg/m^2^ as the cuts of point value and parameter to diagnose acute undernutrition among pregnant women (Abraham, Miruts & Shumye, 2015) and used a cross-sectional design which is not fit to identify determinant of acute undernutrition among pregnant women. Overall, despite the higher burden of maternal undernutrition reported, evidence determined the major risk factors of maternal undernutrition during pregnancy were limited in rural eastern Ethiopia. Given that, it is important to understand the determinant factors affecting the nutritional status of pregnant women in a different direction. Therefore, this study revealed determinants of acute undernutrition among pregnant women attending primary health care units in Chinaksen District, eastern Ethiopia.

## Materials & Methods

### Study design and setting

A facility-based case-control study was conducted from February 01 to March 30, 2017, in Chinaksen District. Chinaksen District is one of the 20 districts in East Hararghe Zone, located 659 kilometers east of Addis Ababa the capital city of Ethiopia. According to the national emergency nutrition coordination unit (ENCU) report of 2015, the district is one of the malnutrition hotspots priority one with semi-pastoral communities in rural eastern Ethiopia. Administratively, the district has 49 rural and three urban kebeles. In 2016, the district had an estimated total population of 119,123, 26,217 women in the reproductive age group, and 4,284 pregnant women (Chinaksen woreda demographic health annual reports of 2016; Chinaksen District Health Office, 2017, unpublished data). According to the District Health Office annual report of 2016, there were eight health centers and 49 health posts providing routine healthcare services for the general public and routine screening of acute undernutrition among pregnant and lactating women since January 2015. Accordingly, on average, 2,096 pregnant women with visible pregnancy were screened monthly for acute undernutrition in 2016, and around 45% of screened pregnant women were undernourished in the district (Chinaksen woreda demographic health annual reports of 2016; Chinaksen District Health Office, 2017, unpublished data).

### Population and sampling

Pregnant women who attended antenatal care follow-up at randomly selected primary healthcare units in Chinaksen District were the study population. Pregnant women with a gestational age of greater than or equal to 20 weeks’ size of fundal height during the data collection period and permanent residents of the district were included in the study. Pregnant women with physical deformities of extremities (paralyzed or fractured or burned or wounded or amputated), critically sick and mentally ill cases/controls who could not respond to interviews were excluded from the study. Cases were pregnant women who attended antenatal care visits in randomly selected primary healthcare units of Chinaksen District and had acute undernutrition (mid-upper-arm-circumference (MUAC) of less than 23 cm) and controls were pregnant women who attended antenatal care visits in the same health facility to cases and had no acute undernutrition (MUAC ≥23 cm) during data collection (Federal Ministry of Health, 2012; Tang et al., 2013; Ververs et al., 2013).

The sample size (*n* = 226) was calculated by Epi-Info version 7.2 software using two population proportion formulas (unmatched case-control study) with the following assumptions (Fleiss, Levin & Paik, 2013; Kasiulevičius, Sapoka & Filipavičiūte, 2016): confidence level of 95%, power of 80%, a margin of error of 5%, one to one controls to cases ratio, 36.1% proportion of exposed controls, adjusted odds ratio (AOR) of 2.32 (Argaw et al., 2015) and 10% non-response proportion. Accordingly, the minimum sample size required for the study was 226 (113 cases and 113 controls).

In this study first, four (Chinaksen, Walensu, Mullisa and Kalaroga) out of eight primary healthcare units of the district were randomly selected using a lottery method. Then, the estimated sample size was allocated proportionally to each selected facility based on the average monthly clients’ flows (pregnant women who attended ANC and screened for acute undernutrition). Accordingly, the average monthly clients’ flow of Chinaksen, Walensu, Mullisa, and Kalaroga primary healthcare units were 231, 150, 170 and 115, respectively, and hence, we allocated a total of 38 cases and 38 controls, 25 cases and 25 controls, 30 cases and 30 controls, and 20 cases and 20 controls to Chinaksen, Walensu, Mullisa, and Kalaroga primary healthcare units, respectively. Then, first, an eligible case was recruited and interviewed, followed by an eligible control (yet two cases or controls not enrolled consecutively), and each day an equal number of cases and controls were enrolled until the desired sample size was fulfilled in each facility.

### Data collection tools and measurements

Data were collected from cases and controls using pretested structured questionnaires adapted from relevant published literature (Abraham, Miruts & Shumye, 2015; Acharya, Bhatta & Timilsina, 2017; Alemayehu, Argaw & Gebramariam, 2015; Coates, Swindale & Bilinsky, 2007; FAO & FHI 360, 2016; Federal Ministry of Health, 2012; Kedir, Berhane & Worku, 2016; Tang et al., 2013) and UNICEF’s three-colored MUAC tape. The question contains information on socio-demographic-related characteristics, reproductive factors, dietary factors, healthcare-related factors and behavioral factors, and the nutritional status of study participants (case/control).

#### Acute undernutrition

It was measured using anthropometric measurement of the women’s left arm at the mid-point between the tip of the shoulder and the tip of the elbow and the insertion type of MUAC tape was non-elastic and non-stretchable to measure with not too loose or too tight with the nearest 0.1 cm reading. The participants considered a case when their MUAC < 23 cm and control when their MUAC ≥ 23 cm and attended the same facility to cases (Federal Ministry of Health, 2012; Tang et al., 2013; Ververs et al., 2013).

#### Decision-making autonomy

It was measured by four dichotomous (yes/no) questions asking about the household decision-making autonomy of women and each item is coded ‘1’ when responded ‘yes’ and coded ‘0’ when responded ‘no’. Then, a composite index score was computed from four items, and the participants had low decision-making autonomy when scored ‘0’, medium decision-making autonomy when scored ‘1-3’ and high decision-making autonomy when scored ‘4’ (Kedir, Berhane & Worku, 2016; Medhin et al., 2010; Tebekaw, 2011).

#### Prenatal dietary advice

It was measured using three dichotomous (yes/no) items each coded to ‘1’ (when responded yes) and to ‘0’ (when responded no) and then, a composite index score was computed from three items, and the prenatal dietary advice considered ‘yes’ when all items responded ‘yes’ and ‘no’ unless otherwise (Kedir, Berhane & Worku, 2016; Tette et al., 2016).

#### Minimum dietary diversity of women (MDDW)

It was measured using ten dichotomous (yes/no) items (asking about the food group consumed by pregnant women in the previous 24 h) coded ‘1’ (when consumed a given food group) and ‘0’ (when not consumed a given food group). Then, a composite index score was computed from 10 items (grain and white roots and tubers and plantains, pulses, nuts and seeds, dairy, meat and poultry and fishes, eggs, dark green leafy vegetables, other vitamin A rich fruits and vegetables, other vegetables, other fruits) and MMDW considered ‘high’ when participant consumed at least five food groups and ‘low’ unless otherwise (FAO & FHI 360, 2016).

#### Household food insecurity

It was measured using nine standard household food insecurity access scale items (Coates, Swindale & Bilinsky, 2007) asking about food access in the household in the last 28 days. Each of the nine items asking the occurrence in households in the last 28 days and coded ‘0’ when responded ‘no’ to an occurrence, ‘1’ when responded ‘rarely’ (occurred 1-2 times), ‘2’ when responded ‘sometimes’ (occurred 3-10 times) and to ‘3’ when responded ‘often’ (occurred 11 times and above). Then, a composite index score was computed from the nine items and it was dichotomous to ‘secure’ when the participants scored zero out of 27 and ‘insecure’ when scored at least one (Abraham, Miruts & Shumye, 2015; Becquey et al., 2010; Gebreyesus et al., 2015).

### Data quality control

To maintain the data quality using standard questionnaires adapted from validated scales and relevant published literature. The questionnaire first prepared in English was translated into local languages (Afaan Oromoo) and back to English by two experts with good command of both languages. We pretested adapted questionnaires on 5% of the total sample (six cases and six controls) to check its validity in a separate non-selected facility (Orda PHCU) in the district and changes were made accordingly. During data collection work, strict supervision of data collectors and validation of collected data was carried out by two supervisors and the principal investigator. Before starting the statistical analysis, several composite index scores were computed and used accordingly that could improve the validity of the measurements and respective computed indices and estimates used in the study (O’brien, 2007; Van der Heijde et al., 1992).

### Data processing and analysis

After checking for completeness and consistency, data were entered into EpiData version 3.1 and analyzed using SPSS version 24. Descriptive statistics such as frequency, a measure of central tendency, and a measure of dispersions were used to characterize case and control populations accordingly. Before analysis, the internal consistencies of items were checked for each composite index score using reliability analysis (Cronbach *α*) and computed the summary statistics, mean (±SD), minimum and maximum scores, and standard error. We observed high internal consistency across all composite indexes with the minimum in decision-making autonomy use items (Cronbach’s *α* = 0.76) and the maximum in prenatal dietary advice use items (Cronbach’s *α* = 0.97) (File S1).

Bivariable and multivariable logistic regression analyses were conducted to identify determinants of acute undernutrition among pregnant women. Multivariable binary logistic regression analyses fitted to determine significant risk factors for acute undernutrition using a backward stepwise LR method of model building and the overall adequacy of the most parsimonious model used for interpretation was confirmed by Hosmer and Lemeshow goodness of fit test (*p*-value > 0.05). Adjusted odds ratio (AOR) with its 95% confidence interval (CI) was used to report the strength of an association, and the statistical significance was declared at *p*-value < 0.05.

### Ethical approval and informed consent

The study was conducted following the Helsinki Declaration of Research involving human subjects (CIOMS & WHO, 2008). The study was approved by the Institutional Review Board (IRB), Institute of Health Sciences, Jimma University, Jimma, Ethiopia (Ref.no: IHRPGC/229/07). Written informed consent was obtained from all participants after explaining the purpose and benefits of the study.

## Results

### Characteristics of participants

A total of 113 cases and 113 controls participated in the study. The majority, (66.4%) of cases and (54.0%) of controls were rural residents. The mean (±SD) age of cases and controls were 26.6 (±5.7) and 28 (±5.5) years respectively. More than half (53.1%) of cases and almost half, (49.6%) of controls were in the age group of 25–34 years old. Seventy-two (63.7%) of cases and (55.8%) of controls had no formal education. The median family size and interquartile range (IQR) of cases and controls were 5 (IQR = 3; 25th percentile = 4, 75th percentile = 7) and 3 (IQR = 3; 25th percentile = 2 and 75th percentile = 5), respectively. Thirty-seven (32.7%) cases and 20.4% of controls had low household decision-making autonomy (Table 1).

More than half (54.9%) of cases and nearly three-fourths (72.6%) of controls had the current pregnancy intention. Fifty-three (46.9%) cases and 36 (31.9%) controls not attended antenatal care during the current pregnancy. Almost three-fourths (74.2%) of cases and half (51.3%) of controls not participated in cooking demonstrations at a kebele level. Fourty-seven (41.6%) of cases and 88 (77.9%) of controls received prenatal dietary advice. The majority (72.6%) of cases and (39.8%) of controls consumed less than five food groups in the last 24 h and their median minimum dietary diversity and IQR of cases and controls were three (IQR = 3; 25th percentile = 2, 75th percentile = 5) and six (IQR = 6; 25th percentile = 3, 75th percentile = 9), respectively. More than half, (58.4%) of cases and one-third (33.6%) of controls were in household food insecure families (Table 2).

**Table 1:** Socio-demographic characteristics of participants in Chinaksen District, eastern Ethiopia, 2017 (*n* = 226).

**Characteristics**		**Cases (%)**	**Controls (%)**
Residence area	Rural	75 (66.4)	61 (54.0)
	Urban	38 (33.6)	52 (46.0)
Age (in year)	<25	40 (35.4)	35 (31.0)
	25–34	60 (53.1)	56 (49.6)
	≥35	13 (11.5)	22 (19.5)
Religion	Muslims	106 (93.8)	100 (88.5)
	Orthodox	6 (5.3)	11 (9.7)
	Protestant	1 (0.9)	2 (1.8)
Ethnicity	Oromo	98 (86.7)	88 (77.9)
	Amhara	3 (2.7)	8 (7.1)
	Somale	9 (8.0)	14 (12.4)
	Guraghe	3 (2.7)	3 (2.7)
Marital status	Married	109 (96.5)	113 (100)
	Others^**a**^	4 (3.5)	0 (0.0)
Educational level	No formal education	72 (63.7)	63 (55.8)
	Primary (grade 1-8)	29 (25.7)	35 (30.9)
	Secondary (grade 9-12) and above	12 (10.6)	15 (13.3)
Main occupation	Unemployed	1 (0.9)	0 (0.0)
	Housewife	80 (70.8)	83 (73.5)
	Merchant	21 (18.6)	15 (13.3)
	Employed	9 (8.0)	14 (12.4)
	Other^**b**^	2 (1.8)	1 (0.9)
Family size	<4	12 (10.6)	57 (50.4)
	≥4	101 (89.4)	56 (49.6)
Average monthly income (in birr)	≤1000	49 (43.4)	31 (27.4)
	>1000	64 (56.6)	82 (72.6)
Polygamy marriage	Yes	17 (15.0)	6 (5.3)
	No	96 (85.0)	107 (94.7)
Intra household violence	Yes	56 (49.6)	28 (24.8)
	No	57 (50.4)	85 (75.2)
Decision making autonomy	Low	37 (32.7)	23 (20.4)
	Medium	62 (54.9.4)	55 (48.7)
	High	14 (12.4)	35 (31.0)

**Notes.**

a divorced or widowed.

b student or daily laborer.

### Determinants of acute undernutrition

In bivariable analysis, residence area, age, family size, average monthly income, polygamy marriage, decision-making autonomy, current pregnancy intention, ANC attendance, prenatal dietary advice, participation in HDA meeting, participation in cooking demonstrations, substance use, basic toilet, minimum dietary diversity of women, and household food security status were significant determinants of acute undernutrition of pregnant women (Table 3).

**Table 2:** Reproductive, healthcare and dietary related characteristics of participants in Chinaksen District, eastern Ethiopia, 2017 (*n* = 226).

**Characteristics**		**Cases (%)**	**Controls (%)**
Gravidity	<5	77 (68.1)	79 (69.9)
	≥5	36 (31.9)	34 (30.1)
Parity	<5	96 (85.0)	91 (80.5)
	≥5	17 (15.0)	22 (19.5)
Previous abortion	Yes	15 (13.3)	10 (8.8)
	No	98 (86.7)	103 (91.2)
Current pregnancy intention	Planned	62 (54.9)	82 (72.6)
	Unplanned	51 (45.1)	31(27.4)
Contraceptives utilization	Yes	39 (34.5)	53 (46.9)
	No	74 (65.5)	60 (53.1)
Antenatal care attendance	Yes	60 (53.1)	77 (68.1)
	No	53 (46.9)	36 (31.9)
Time to reach nearby health facility on foot	>30 Minute	39 (34.5)	54 (47.8)
	≤30 Minute	74 (65.5)	59 (52.2)
Participated on HDA meeting	Yes	34 (30.1)	48 (42.5)
	No	79 (69.9)	65 (57.5)
Participated on cooking demonstration	Yes	28 (24.8)	55 (48.7)
	No	85 (74.2)	58 (51.3)
Prenatal dietary advice	Yes	47 (41.6)	88 (77.9)
	No	66 (58.4)	25 (22.1)
Substance use (at least one)	Yes	102 (90.3)	92 (81.4)
	No	11 (9.7)	21 (18.6)
Basic toilet possession	Yes	63 (55.8)	93 (82.3)
	No	50 (44.2)	20 (17.7)
Main source of drink water	Protected	40 (35.4)	51 (45.1)
	Unprotected	73 (64.6)	62 (54.9)
Minimum dietary diversity of women	Low	82 (72.6)	45 (39.8)
	High	31 (27.4)	68 (60.2)
Household food security status	Secure	47 (41.6)	75 (66.4)
	Insecure	66 (58.4)	38 (33.6)

**Notes.**

HDA Health Development Army

**Table 3:** Determinants of acute undernutrition among pregnant women in Chinaksen District, eastern Ethiopia, 2017 (*n* = 226).

**Determinants**		**Cases (%)**	**Controls (%)**	**COR (95% CI)**	***p*-value**	**AOR (95% CI)**	***p*-value**
Residence area	Urban	75 (66.4)	61 (54.0)	1			
	Rural	38 (33.6)	52 (46.0)	1.68 (0.98, 2.88)	0.058	0.71 (0.30, 1.68)	0.490
Age (in year)	<25	40 (35.4)	35 (31.0)	1.93 (0.85, 4.40)	0.116	2.24 (0.72, 7.82)	0.166
	25-34	60 (53.1)	56 (49.6)	1.81 (0.83, 3.94)	0.133	0.95 (0.28, 2.94)	0.943
	≥35	13 (11.5)	22 (19.5)	1			
Family size	<4	12 (10.6)	57 (50.4)	1			
	≥ 4	101 (89.4)	56 (49.6)	8.57(4.24,17.30)	<0.001	6.98 (2.82, 17.27)	<0.001
Average monthly income (in birr)	≤1000	49 (43.4)	31 (27.4)	2.02 (1.16, 3.53)	0.013	1.26 (0.52, 3.05)	0.651
	>1000	64 (56.6)	82 (72.6)	1			
Polygamy marriage	Yes	17 (15.0)	6 (5.3)	3.16 (1.20, 8.33)	0.020	1.31 (0.39, 4.37)	0.649
	No	96 (85.0)	107 (94.7)	1			
Decision making autonomy	Low	37 (32.7)	23 (20.4)	4.02 (1.79, 9.03)	0.001	2.07 (0.69, 6.17)	0.181
	Medium	62 (54.9)	55 (48.7)	2.82 (1.37, 5.78)	0.005	1.78 (0.68, 4.67)	0.221
	High	14 (12.4)	35 (31.0)	1			
Pregnancy intention	Planned	62 (54.9)	82 (72.6)	1			
	Unplanned	51 (45.1)	31 (27.4)	2.18 (1.25, 3.40)	0.006	1.33 (0.60, 2.92)	0.503
Antenatal care attend	Yes	60 (53.1)	77 (68.1)	1			
	No	53 (46.9)	36 (31.9)	1.89 (1.10, 3.25)	0.021	0.73 (0.31, 1.72)	0.500
Prenatal dietary advice	Yes	47 (41.6)	88 (77.9)	1			
	No	66 (58.4)	25 (22.1)	4.94 (2.77, 8.83)	<0.001	3.68 (1.70, 8.00)	<0.001
Participated on HDA meeting	Yes	34 (30.1)	48 (42.5)	1			
	No	79 (69.9)	65 (57.5)	1.72 (0.99, 2.97)	0.054	0.92 (0.41, 2.11)	0.781
Participated on cooking demonstration	Yes	28 (24.8)	55 (48.7)	1			
	No	85 (74.2)	58 (51.3)	2.88 (1.64, 5.06)	<0.001	5.41 (2.39, 12.24)	<0.001
Substances use (at least one)	Yes	102 (90.3)	92 (81.4)	2.12 (0.97, 4.63)	0.060	3.65 (1.30, 10.23)	0.013
	No	11 (9.7)	21 (18.6)	1			
Basic toilet possession	Yes No	63 (55.8) 50 (44.2)	93 (82.3) 20 (17.7)	1 3.69 (2.01, 6.79)	<0.001	2.91 (1.28, 6.58)	0.014
Minimum dietary diversity of women	Low	82 (72.6)	45 (39.8)	4.00 (2.28, 6.99)	<0.001	2.48 (1.20, 5.12)	0.012
	High	31 (27.4)	68 (60.2)	1			
Household food security status	Secure	47 (41.6)	75 (66.4)	1			
	Insecure	66 (58.4)	38 (33.6)	2.77 (1.61, 4.76)	<0.001	3.06 (1.44, 6.51)	0.003

**Notes.**

COR Crude Odds Ratio AOR Adjusted Odds Ratio CI Confidence Interval HDA Health Development Army

In the multivariable analysis, large family size (AOR = 6.98, 95% CI [2.82–17.27]), lack of prenatal dietary advice (AOR = 3.68, 95% CI [1.67–8.00]), did not participate in cooking demonstrations (AOR = 5.41, 95% CI [2.39–12.24]), used substances (AOR = 3.65, 95% CI [1.30–10.23]), absent of basic toilet (AOR = 2.91, 95% CI [1.28–6.58]), low minimum dietary diversity of women (AOR = 2.48, 95% CI [1.20–5.12]), and household food insecurity (AOR = 3.6, 95% CI [1.44–6.51]) were significant risk factors of acute undernutrition of pregnant women (Table 3).

## Discussion

As the burden of maternal undernutrition during pregnancy is significantly high in SSA in general and rural eastern Ethiopia (Branca et al., 2015; Central Statistical Agency Ethiopia and ICF, 2016; Kedir, Berhane & Worku, 2016; WHO, 2017), massive risk factors affecting maternal nutritional status (Desalegn et al., 2015; Kedir, Berhane & Worku, 2016; Mathewos, Amare & Eskindir, 2015; Nigatu, Gebrehiwot & Gemeda, 2018). It would be important to understand the major preventable risk factors for acute undernutrition during pregnancy in different circumstances. Therefore, this study determined the major risk factors for acute undernutrition of pregnant women in rural eastern Ethiopia.

This study showed that living in crowded family, did not receive prenatal dietary advice, did not participate in cooking demonstrations, substances used, absence of a basic toilet, having low minimum dietary diversity, and household food insecurity were statistically significant risk factors for acute undernutrition of pregnant women.

This study revealed that the odds of acute undernutrition were higher among pregnant women in large family sizes than those in small family sizes. This finding was supported by the studies conducted in southeast Amhara, Ethiopia (Tariku et al., 2014), western Ethiopia (Ayana, Hailemariam & Melke, 2015), and Malaysia (Wong, Moy & Nair, 2014) indicating that large family size is a major risk factor for acute undernutrition. This implies that large family sizes increase the risk factors for acute undernutrition among pregnant women. This could be due to pregnant women in large family sizes being more likely to expense energy due to the workload of care for their family. In addition, large family sizes might affect the feeding quality and frequency of pregnant women.

This study indicated pregnant women who did not receive prenatal dietary advice were four times more likely undernourished than those who received the advice. This finding was in agreement with the studies conducted in southwest Ethiopia (Shiferaw & Husein, 2019), eastern Ethiopia (Kedir, Berhane & Worku, 2016), and Bangladesh (Nguyen et al., 2017). This could imply that prenatal dietary advice is one of the key nutritional interventions that improve the nutritional status of pregnant women (Diddana et al., 2018). It recommended that all health professionals should provide dietary diversity advice at every contact of women with health services.

The finding of this study showed pregnant women who did not participate in cooking demonstrations at a kebele level were about five times more likely malnourished than women who participated in the demonstration.

The study also indicated odds of acute undernutrition were higher among pregnant women who used substances compared to those who did not use the substances. This finding was supported by the studies conducted in eastern Ethiopia (Kedir, Berhane & Worku, 2013), southwest Ethiopia (Shiferaw & Husein, 2019), and the study conducted in Pakistan (Ali et al., 2020). This could be due to the biological fact that substances use causes poor appetite and gastritis which leads to low food consumption and poor nutrient absorption.

The odds of acute undernutrition were also higher among pregnant women with lack of a basic toilets than their counterparts. This finding was in agreement with the studies conducted in east Hararghe, eastern Ethiopia (Abebaw & Zewdie, 2013), Jimma, southwest Ethiopia (Shiferaw & Husein, 2019), west Oromia, western Ethiopia (Ayana, Hailemariam & Melke, 2015), southeast Amhara, Ethiopia (Tariku et al., 2014), and Iran (Sharghi, Kamran & Faridan, 2011).

In addition, pregnant women who had low dietary diversity scores were three times more likely to be undernourished compared to those who had better scores. This finding was inconsistence with the studies conducted in Jimma, southwest Ethiopia (Alemayehu, Argaw & Gebramariam, 2015), Dessie, northeast Ethiopia (Seid & Cherie, 2022), and Nepal (Lama et al., 2018). This implies that feeding monotonous food with insufficient macro and micronutrients nutritionally vulnerable pregnant women (FAO & FHI 360, 2016). This could be due to sufficient macro and micronutrients being essential for enhancing women’s immunity and metabolic activities during pregnancy.

Furthermore, pregnant women from food-insecure households were three times more likely to be undernourished compared to their counterparts. This finding was supported by the studies conducted in Jimma, southwest Ethiopia (Shiferaw & Husein, 2019); Tigray, northern Ethiopia (Abraham, Miruts & Shumye, 2015); Gambella, western Ethiopia (Nigatu, Gebrehiwot & Gemeda, 2018) and Nepal (Acharya, Bhatta & Timilsina, 2017). Household food insecurity is one of the underline causes of undernutrition, which exists when the household at all times has no physical, social and economic access to sufficient, safe, and nutritious food that meets their dietary needs for an active and healthy life. This could be due to the time of short food supply, as women consume less food relative to men. In addition, during a shortage of food, the women used coping strategies to reduce their food intake and to secure food for their infants and small children. Given that, improving household food security in the community is important to reduce and prevent acute undernutrition and its long-term negative effects.

Applying a case-control study design to determine the determinants of acute undernutrition among pregnant women is a strength of the study. The study limitation, since some of the questionnaires were asking about the events 24 h and four weeks back, recall bias might affect the true relations between exposure and disease. In addition, the causal relationship between variables was not studied.

## Conclusions

This study revealed that crowded families, lack of awareness and skill of dietary diversity, low quality and quantity/access of food, poor hygiene and sanitation, and substance use were independent risk factors of acute undernutrition of pregnant women. Strengthening multi-sectoral approaches through improving dietary diversity/quality and food security/access in the community would be essential to prevent and reduce the burden and risk factors of acute undernutrition during pregnancy and its negative long-term consequences. In addition, providing behavioral change nutritional counseling supported by demonstration and protecting individual and environmental hygiene of the community would be needed to prevent acute undernutrition of pregnant women.

##  Supplemental Information

10.7717/peerj.15416/supp-1 Supplemental Information 1 Summary statistics of composite, determinants of acute undernutrition among pregnant women in Chinaksen district, eastern Ethiopia, 2017 (*n* = 216)

10.7717/peerj.15416/supp-2 Supplemental Information 2 Determinants of Acute Undernutrition among Pregnant Women attending Primary Healthcare Unit in Chinaksen District, Eastern Ethiopia SPSS data

10.7717/peerj.15416/supp-3 Supplemental Information 3 Questionnaire, Determinants of Acute Undernutrition among Pregnant Women

## Abbreviations

AOR: Adjusted Odds Ratio
HDA: Health Development Army
MDDW: Minimum Dietary Diversity for women
PHCU: Primary Health Care Unit
SSA: Sub-Saharan Africa
WHO: World Health Organization

## Additional Information and Declarations

### Data Availability

The following information was supplied regarding data availability: The raw data is available in the Supplemental Files.
