# Supplementary material for: Determinants of acute undernutrition among pregnant women attending primary healthcare unit in Chinaksen District, Eastern Ethiopia: a case-control study
Source: PeerJ. 2023 Jun 5;11:e15416. doi: 10.7717/peerj.15416 (PMC10249615; doi:10.7717/peerj.15416)
Supplement: Supplemental Information 1 [file peerj-11-15416-s001.docx]

**Supplementary file 1**

**Summary statistics of composite determinants of acute undernutrition among pregnant women in Chinaksen district, eastern Ethiopia, 2017 (n=216)**

| **Composite indices** | **Number of items** | **Cronbach’s**  **α** | **Minimum** | **Maximum** | **Mean** | **SD** | **SE** |
| --- | --- | --- | --- | --- | --- | --- | --- |
| Decision making autonomy | 4 | 0.76 | 0 | 4 | 1.88 | 1.48 | 0.10 |
| Prenatal dietary advice | 3 | 0.97 | 0 | 3 | 1.88 | 1.41 | 0.06 |
| Minimum dietary diversity of women | 10 | 0.82 | 0 | 10 | 4.77 | 2.57 | 0.17 |
| Household food security status | 10 | 0.88 | 0 | 14 | 2.30 | 3.17 | 0.21 |
