# Supplementary material for: Determinants of acute undernutrition among pregnant women attending primary healthcare unit in Chinaksen District, Eastern Ethiopia: a case-control study
Source: PeerJ. 2023 Jun 5;11:e15416. doi: 10.7717/peerj.15416 (PMC10249615; doi:10.7717/peerj.15416)
Supplement: Supplemental Information 3 [file peerj-11-15416-s003.docx]

**Questionnaires**

| **Questionnaire Identification Data** | | | |
| --- | --- | --- | --- |
|  | Participants ID | | __________________ |
|  | Date of interview (DD/MM/YYYY) | | ______________ |
|  | Name of Health Facility (PHCU) | | _____________ |
|  | Checked by Supervisor | _**_______________ ____________ ____________** ^Name Signature Date^ | |

| **Part I: Socio-demographic characteristics** | | |
| --- | --- | --- |
| **No** | **Question** | **Responses** |
| 1 | Current residency area | 0. Urban 1. Rural |
| 2 | Age in complete year | ___________year |
| 3 | Religion | 1.Muslim  2. Orthodox  3.Protestant,  4. Others (specify)____ |
| 4 | Ethnicity | 1.Oromo  2.Amhara  3.Somale  4.Other(specify)______ |
| 5 | Marital status | 1.Married,  2. Divorced  3.Widowed,  4.Separated |
| 6 | Educational status | 1. unable to read and write  2. able to read and write  3. Primary School (grade 1-8)  4. Secondary School (Grade 9-12)  5. College and above |
| 7 | Husband Educational status | 1. unable to read and write  2. able to read and write  3. Primary School (grade 1-8)  4. Secondary School (Grade 9-12)  5. College and above |
| 8 | Current main occupation | 1. Unemployed  2. Housewife  3.Merchant  4.Employee  5.Others (specify)_________ |
| 9 | Husband current main occupation | 1. Unemployed  2. Housewife  3.Merchant  4.Employee  5.Others (specify)__________ |
| 10 | Are your family/HH is/are the general (food/money) aids (of any type) during current pregnancy? | 0.Yes  1.No |
| 11 | Family sizes/person living house | ___________ |
| 12 | Average monthly income (in ETB) | __________birr |
| 14 | Polygamy Marriage | 0.No  1.Yes |
| 15 | Do you have history of (at least once) intra household violence practice in the family? | 0.No  1.Yes |
| 16 | Do you have autonomy of your own on seeking the health care /treatment from health facilities? | 0.No  1.Yes |
| 17 | Do you have autonomy of your own on the major household purchase in/for your family? | 0.No  1.Yes |
| 18 | Do you have autonomy of your own on the major household expenditures in/for your family? | 0.No  1.Yes |
| 19 | Do you have an autonomy of your own on visiting of friends/relative or participate on any meeting | 0.No  1.Yes |
| 20 | Weight (in Kilograms) | __________Kg |
| 21 | Height (in Centimeters) | __________cm |
| 22 | MUAC? (in Centimeters) | ________cm |

| **Part II: Reproductive and health care related factors** | | |
| --- | --- | --- |
| **No** | **Question** | **Responses** |
| 1 | Gravidity | ________ |
| 2 | Parity | ________ |
| 3 | Number of previous abortions | _______ |
| 4 | What is (your) intention for (your) current pregnancy? | 1. planned  0. unplanned |
| 5 | Did you ever use contraceptive methods (of any type/s)? | 1. Yes  0. No |
| 6 | Did you ever visited health facilities for ANC services during current pregnancy? | 1. Yes  0. No |
| 7 | How many times did you visit ANC service including your current visit? | ______times |
| 8 | Average walking time to reach your nearby public Health facilities in minute | _____minute |
| 9 | What is your main/usual source of drinking water | 1. protected  0. unprotected |
| 10 | Did you screened for (acute) malnutrition (maternal wasting) by MUAC tape during current/previous pregnancy? | 0. Yes  1. No |
| 11 | Do you ever participated on health development army meeting in kebele? | 0. Yes  1. No |
| 12 | Do you ever participated on complementary feeding demonstration in kebele? | 1. Yes  0. No |
| 13 | Do you ever advised (at least once by HEW/HW/MD) to eat more diet/meals during current pregnancy? | 1. Yes  0. No |
| 14 | Do you ever advised (at least once by HEW/HW/MD) to eat balanced diets during your current pregnancy? | 1. Yes  0. No |
| 15 | Do you ever advised (at least once by HEW/HW/MD) to eat different fruits/vegetables during your current pregnancy? | 1. Yes  0. No |
| 16 | Do you ever chewed khat or smoked cigarettes or drunken alcohols (at least once) during current pregnancy? | 1. Yes  0. No |
| 17 | Basic latrine available | 1.Yes  0.No |
| 18 | Hands washing after toilet (with soap and or ashes) | 1.Yes  0.No |

| **Part III: Minimum Dietary Diversity of women (MDD-W) model questionnaire** | | | | |
| --- | --- | --- | --- | --- |
| **Required 14 rows will be aggregated during analysis into the 10 MDD-W food groups** | | | | |
| No | **Food categories** | **Examples** | | **Consumed**  **Yes = 1 No= 0** |
| 1 | **Foods made from** **grains** | *Porridge, bread, rice, pasta/noodles or other foods made from grains* | | ______ |
| 2 | **White roots** **and tubers and** **plantains** | *White potatoes, white yams, manioc/cassava/yucca, cocoyam, taro or* *any other foods made from white fleshed roots or tubers, or plantains* | | ______ |
| 3 | **Pulses (beans, peas** **and lentils)** | *Mature beans or peas (fresh or dried seed), lentils or bean/pea products,* *including hummus, tofu and tempeh* | | ­­  _______ |
| 4 | **Nuts and seeds** | *Any tree nut, groundnut/peanut or certain seeds, or nut/seed “butters”* *or pastes* | | ________ |
| 5 | **Milk and milk** **products** | *Milk, cheese, yoghurt or other milk products but NOT including butter,* *ice cream, cream or sour cream* | | ______ |
| 6 | **Organ meat** | *Liver, kidney, heart or other organ meats or blood-based foods,* *including from wild game* | | ______ |
| 7 | **Meat and poultry** | *Beef, pork, lamb, goat, rabbit, wild game meat, chicken, duck or other* *bird* | | ______ |
| 8 | **Fish and seafood** | *Fresh or dried fish, shellfish or sea food* | | ______ |
| 9 | **Eggs** | *Eggs from poultry or any other bird* | | ______ |
| 10 | **Dark green leafy** **vegetables** | *List examples of any medium-to-dark green leafy vegetables, including* *wild/foraged leaves* | | ______ |
| 11 | **Vitamin A-rich** **vegetables, roots** **and tubers** | *Pumpkin, carrots, squash or sweet potatoes that are yellow or orange* | | ______ |
| 12 | **Vitamin A-rich** **fruits** | *Ripe mango, ripe papaya, vitamin A-rich fruits* | | ______ |
| 13 | **Other vegetables** | *List examples of any other vegetables* | | ______ |
| 14 | **Other fruits** | *List examples of any other fruits* | | ______ |
| ***Other food categories, not included in construction of MDD-W*** | | | | |
| 15 | **Oils And Fats** | *Oil, fats or butter added to food or used for cooking (oil in sauce)* | ______ | |
| 16 | **Sweet** | *sugar, honey, sweetened, soda, sweetened, juice or sugary foods such as chocolates, candies, cookies and cake (sugar in tea)* | ______ | |
| 17 | **Spices**  **Condiments**  **Beverages** | *spices like black pepper, salt and condiments like powder and tea, condiments (soy sauce, hot sauce), coffee, tea, alcohols and beverages (beverages or local examples)* |  | |
| **Required** | | | | |
| 18 | **Condiments and** **seasonings** | *Ingredients used in small quantities for flavour, such as chilies, spices,* *herbs, fish powder, tomato paste, flavour cubes or seeds* | ______ | |
| 19 | **Other beverages** **and foods** | *Tea or coffee if not sweetened, clear broth, alcohol* *Pickles, olives and similar* | ______ | |

| **Part IV: Household Food Insecurity Access Scale (HFIAS) Tool** | | | |
| --- | --- | --- | --- |
| **No** | **Questions** | **Response Choices** | **CODE (answer)** |
| **101** | In past 4 weeks, did you worry that your House Hold (HH) would not have enough food? | 0 = No (skip to Q102) 1=Yes | ___ |
| **101a** | How often did this happen? | 1 = Rarely (1-2times in past 4wks)  2 = Sometime (3-10times “ “ ) 3 = Often (≥ 11times in past 4wks) | ___ |
| **102** | In past 4 wks, were you or any HH members not able to eat the kinds of foods you preferred due to of a lack of resources? | 0 = No (skip to Q103) 1=Yes | ___ |
| **102a** | How often did this happen? | 1 = Rarely (1-2times in past 4wks)  2 = Sometime (3-10times “ “ ) 3 = Often (≥ 11times in past 4wks) | ___ |
| **103** | In past 4 wks, did you or any HH member have to eat limited variety of foods due to lack of money? | 0 = No (skip to Q104) 1 = Yes | ___ |
| **103a** | How often did this happen? | 1 = Rarely (1-2times in past 4wks)  2 = Sometime (3-10times “ “ ) 3 = Often (≥ 11times in past 4wks) | ___ |
| **104** | In past 4 weeks, did you or any HH member have to eat some foods that you really didn’t want to eat due to lack of money to get other food type? | 0 = No (skip to Q105) 1 = Yes | ___ |
| **104a** | How often did this happen? | 1 = Rarely (1-2times in past 4wks)  2 = Sometime (3-10times “ “ ) 3 = Often (≥ 11times in past 4wks) | ___ |
| **105** | In past 4 wks, did you or any HH member have to eat smaller meal than you felt you needed due to no enough food? | 0 = No (skip to Q106) 1 = Yes | ___ |
| **105a** | How often did this happen? | 1 = Rarely (1-2times in past 4wks)  2 = Sometime (3-10times “ “ ) 3 = Often (≥ 11times in past 4wks) | ___ |
| **106** | In past 4 wks, did you or any HH member have to eat fewer meals in day due to no enough food? | 0 = No (skip to Q107) 1 = Yes | ___ |
| **106a** | How often did this happen? | 1 = Rarely (1-2times in past 4wks)  2 = Sometime (3-10times “ “ ) 3 = Often (≥ 11times in past 4wks) | ___ |
| **107** | In past 4 wks, was there ever no food to eat of any kind in your HH due to lack of money to get it? | 0 = No (skip to Q108) 1 = Yes | ___ |
| **107a** | How often did this happen? | 1 = Rarely (1-2times in past 4wks)  2 = Sometime (3-10times “ “ ) 3 = Often (≥ 11times in past 4wks) | ___ |
| **108** | In past 4 wks, did you or any HH member go to sleep at night hungry due to not enough food? | 0 = No (skip to Q109) 1 = Yes | ___ |
| **108a** | How often did this happen? | 1 = Rarely (1-2times in past 4wks)  2 = Sometime (3-10times “ “ ) 3 = Often (≥ 11times in past 4wks) | ___ |
| **109** | In past 4 wks, did you or any HH member go a whole day and night without eating anything due to there was not enough food? | 0 = No (question is ended) 1 = Yes | ___ |
| **109a** | How often did this happen? | 1 = Rarely (1-2times in past 4wks)  2 = Sometime (3-10times “ “ ) 3 = Often (≥ 11times in past 4wks) | ___ |
